# Mitochondria in Ageing and Diseases: The Super Trouper of the Cell

**DOI:** 10.3390/ijms17050711

**Published:** 2016-05-11

**Authors:** Giuseppe Coppotelli, Jaime M. Ross

**Affiliations:** 1 Department of Neuroscience, Karolinska Institutet, Retzius väg 8, Stockholm 171 77, Sweden; 2 Department of Genetics, Harvard Medical School, 77 Avenue Louis Pasteur, Boston, MA 02215, USA

The past decade has witnessed an explosion of knowledge regarding how mitochondrial dysfunction may translate into ageing and disease phenotypes, as well as how it is modulated by genetic and lifestyle factors. In addition to energy production, mitochondria play an important role in regulating apoptosis, buffering calcium release, retrograde signaling to the nuclear genome, producing reactive oxygen species (ROS), participating in steroid synthesis, signaling to the immune system, as well as controlling the cell cycle and cell growth. Impairment of the mitochondria may be caused by mutations or deletions in nuclear or mitochondrial DNA (mtDNA). Hallmarks of mitochondrial dysfunction include decreased ATP production, decreased mitochondrial membrane potential, swollen mitochondria, damaged cristae, increased oxidative stress, and decreased mitochondrial DNA copy number.

Dysfunctional mitochondria have been implicated in ageing and in several diseases, many of which are age-related, including mitochondrial diseases, cancers, metabolic diseases and diabetes, inflammatory conditions, neuropathy, and neurodegenerative diseases such as Alzheimer’s, Parkinson’s, and Huntington’s disease. Additionally, a possible link between mitochondrial metabolism and the ubiquitin-proteasome and autophagy-lysosome systems is emerging as a novel factor contributing to the progression of several human diseases. The purpose of the Special Issue “Mitochondrial Dysfunction in Ageing and Diseases” published in the *International Journal of Molecular Sciences* [1] was to capture reviews, perspectives, and original research articles to address the progress and current standing in the vast field of mitochondrial biology. A total of 21 papers consisting of 17 reviews and 4 articles have been published as part of the Special Issue as detailed in Table 1. Topics included range from mitochondrial function, cell signaling, and protein homeostasis to disorders and diseases where mitochondrial dysfunction are implicated, such as metabolic diseases, ageing, several age-related diseases, as well as cancer. Various therapies to counteract mitochondrial dysfunction are also discussed.

The Special Issue opens with three reviews describing aspects of the mitochondrial machinery in the context of maintaining homeostasis and disease [2,3,4]. Ding and Liu [4] together with Ahmed *et al.* [2] summarize the maintenance and replication of the mitochondrial genome, and discuss how DNA helicases and mtDNA instability affect integrity of the mtDNA, thus contributing to mitochondrial diseases and disorders. Mitochondrial retrograde signaling, specifically the mitochondrial unfolded protein response, involved in proteostasis is reviewed by Arnould *et al.* [3]. The next five papers review the interconnectedness of mitochondrial dysfunction and protein homeostasis in health, ageing, and diseases [5,6,7,8,9]. Ross *et al.* [5] discuss the interplay of mitochondrial dysfunction and impairment of the ubiquitin proteasome system in ageing and disease, and provide a hypothetical model to address the heterogeneity often described during ageing. The heterogeneity of skeletal muscle performance in ageing is examined by Crescenzo *et al.* [6], taking into account the diverse mitochondrial populations present in skeletal muscle. Tricarico *et al.* [7] focus on a possible link between mitochondrial dysfunction, defective protein prenylation, and the mevalonate pathway, crucial for cholesterol synthesis, with disease. Zhang *et al.* [8] describe under physiological and pathological conditions the modulators of autophagy that regulate erythropoiesis, a process during which mitochondria and other intracellular organelles are removed. The intersection of mtDNA damage and oxidative stress on age-related vascular dysfunction is presented by Mikhed *et al.* [9], with particular focus on nicotinamide adenosine dinucleotide phosphate (NADPH) oxidases.

We received several reviews and research articles implicating mitochondria in cardiovascular diseases and ischemia as well as cerebral hypoxia-ischemia [11,12,13,14]. Interestingly, a few of these contributions highlight the role of thyroid hormone [10,11,12]. Vaikus *et al.* [10] review the diverse effects that thyroid hormone has on mitochondria and energy expenditure, including mitochondrial biogenesis and clinical correlates. Forini *et al.* [12] discuss possible thyroid hormone triiodothyronine (T3) supplementation to improve mitochondrial function in the context of ischemic heart disease. The same research group also present findings [11] indicating that low T3 levels are correlated with mitochondrial impairments following cardiac ischemia reperfusion injury. Mitochondrial dysfunction is also associated with septic cardiomyopathy, a complication of sepsis, which is a serious condition where the pathogenesis and underlying mechanisms remain unclear, as described by Cimolai *et al.* [13]. Findings discussed by Babiramani *et al.* [14] suggest that neonatal cerebral hypoxic-ischemia may alter mitochondrial dynamics, affecting optic atrophy 1 (OPA1). Impaired mitochondrial dynamics have also been described in neurodegenerative diseases, such as Parkinson’s disease (PD). Luo *et al.* [15] review recent literature that support the role of compromised mitochondrial dynamics, mitophagy, and mitochondrial import in PD, and also offer a list of potential therapeutics that target mitochondria. The review by Kim *et al.* [16] discusses the link between mitochondrial dysfunction and alveolar epithelial cell apoptosis in contributing to age-related lung diseases, as well as how sirtuin family members may constitute therapeutic candidates. Mitochondrial co-factors, such as α-lipoic acid, carnitine, and Coenzyme Q10 have been used to treat mitochondria-associated disorders and diseases, and the results of several clinical trials using these co-factors with and without antioxidants/herbal compounds are systematically presented by Pagano *et al.* [17].

A few contributions regarding the involvement of mitochondria in cancer and possible therapies were also received [18,19,20,21]. Ever since the “Warburg effect” was described nearly a century ago, mitochondria have been increasingly implicated in cancer biology. Extensive research has revealed notable differences between cancerous and healthy cells, such as altered mitochondrial size, shape, metabolic profiles, membrane potential, as well as elevated levels of mtDNA mutations, mitochondrial transcription factor A (TFAM), and oxidative stress [18,20]. Using these findings to exploit mitochondria, several promising anti-cancer treatments have been developed, but have unfortunately proven to have limitations [18,19,20,21]. Thus, researchers have recently explored alternative strategies as summarized in Modica-Napolitano and Weissig [18] as well as targeting specific microenvironments within tumors as discussed by Zhang *et al.* [19]. Moreover, the mechanisms by which cancer cells develop drug-resistance are currently being investigated, as reviewed by Kohno *et al.* [20], and possible means to mitigate the side effects of anti-cancer therapies are also being studied by Wang *et al.* [21]. Collectively, these contributions focus on mitochondrial mechanisms as an avenue to reveal possible novel interventions in order to combat cancer.

Lastly, but certainly not of least importance, recent studies of the role of mitochondrial function in fertility and oocyte quality have been extensive. Research by Wang *et al.* [22] demonstrates that androgen receptor knockout mice have poor oocyte maturating rates, impaired ATP production in granulosa cell mitochondria, and impaired mitochondria biogenesis. Additional research is needed to better understand how mitochondrial function may affect fertility and fecundity in order to develop therapeutic approaches.

Overall, the 21 contributions published in the Special Issue illustrate how essential mitochondria are to overall health and success of an organism. The involvement of mitochondria in several biological disciplines, diseases, and disorders, ranging from cancer biology, metabolism, and proteostasis to neurodegenerative and cardiovascular diseases is a testament to their importance and fundamental contributions. We would like to thank all of the authors who contributed their work to the Special Issue. The main objective was to provide ample breadth and depth to depict the interconnectedness of mitochondrial function in ageing and mitochondrial-associated diseases. While the underlying mechanisms linking impaired mitochondria with the ageing process and disease states remain incompletely elucidated, the overall field of mitochondrial biology has made leaps and bounds in only the past two decades. Based on these breakthroughs, new “mito-research” platforms have emerged; for example, mitochondrial function in fertility or in stem cell niches. We remain hopeful that harnessing the power of the mitochondrial network will help us stay healthy.

## Figures and Tables

**Table 1:** Summary of papers in the Special Issue, arranged by topic as pertaining to mitochondrial dysfunction.

Authors	Title	Topics/Keywords	Type
Ahmed *et al.* [2]	Genes and Pathways Involved in Adult Onset Disorders Featuring Muscle Mitochondrial DNA Instability	mtDNA Maintenance; Mitochondrial Disorders	Review
Arnould *et al.* [3]	Mitochondria Retrograde Signaling and the UPRmt: Where Are We in Mammals?	Unfolded Protein Response; Cell Signaling	Review
Ding *et al.* [4]	Borrowing Nuclear DNA Helicases to Protect Mitochondrial DNA	DNA Replication and Repair; Diseases	Review
Ross *et al.* [5]	Mitochondrial and Ubiquitin Proteasome System Dysfunction in Ageing and Disease: Two Sides of the Same Coin?	Ageing and Age-Related Diseases; Ubiquitin; Proteasome	Review
Crescenzo *et al.* [6]	Skeletal Muscle Mitochondrial Energetic Efficiency and Aging	Ageing; Skeletal Muscle	Review
Tricarico *et al.* [7]	Mevalonate Pathway Blockade, Mitochondrial Dysfunction and Autophagy: A Possible Link	Cholesterol Synthesis; Autophagy; Inflammation	Review
Zhang *et al.* [8]	Autophagy as a Regulatory Component of Erythropoiesis	Autophagy; Erythroid Differentiation	Review
Mikhed *et al.* [9]	Mitochondrial Oxidative Stress, Mitochondrial DNA Damage and Their Role in Age-Related Vascular Dysfunction	Ageing; Cardiovascular Diseases; Oxidative Stress	Review
Vaitkus *et al.* [10]	Thyroid Hormone Mediated Modulation of Energy Expenditure	Thyroid Hormone; Energy Expenditure	Review
Forini *et al.* [11]	Low T3 State Is Correlated with Cardiac Mitochondrial Impairments after Ischemia Reperfusion Injury: Evidence from a Proteomic Approach	Thyroid Hormone; Cardiac Ischemia and Reperfusion; Proteomics	Article
Forini *et al.* [12]	Mitochondria as Key Targets of Cardioprotection in Cardiac Ischemic Disease: Role of Thyroid Hormone Triiodothyronine	Cardiac Ischemia; Thyroid Hormone; Cardioprotection	Review
Cimolai *et al.* [13]	Mitochondrial Mechanisms in Septic Cardiomyopathy	Septic Cardiomyopathy; Mitophagy	Review
Baburamani *et al.* [14]	Mitochondrial Optic Atrophy (OPA) 1 Processing Is Altered in Response to Neonatal Hypoxic-Ischemic Brain Injury	Hypoxia-Ischemia; Brain Injury	Article
Luo *et al.* [15]	Mitochondria: A Therapeutic Target for Parkinson’s Disease?	Parkinson’s Disease; Mitochondrial Dynamics	Review
Kim *et al.* [16]	The Role of Mitochondrial DNA in Mediating Alveolar Epithelial Cell Apoptosis and Pulmonary Fibrosis	Pulmonary Fibrosis; Sirtuins	Review
Pagano *et al.* [17]	Current Experience in Testing Mitochondrial Nutrients in Disorders Featuring Oxidative Stress and Mitochondrial Dysfunction: Rational Design of Chemoprevention Trials	Mitochondrial Co-Factors as Therapeutics	Review
Modica-Napolitano *et al.* [18]	Treatment Strategies that Enhance the Efficacy and Selectivity of Mitochondria-Targeted Anticancer Agents	Cancer; Combination Therapy	Review
Zhang *et al.* [19]	Targeting Mitochondrial Function to Treat Quiescent Tumor Cells in Solid Tumors	Cancer; Cancer Therapies	Review
Kohno *et al.* [20]	Mitochondrial Transcription Factor A and Mitochondrial Genome as Molecular Targets for Cisplatin-Based Cancer Chemotherapy	mtTFAM; Cancer Chemotherapy	Review
Wang *et al.* [21]	Mitochondria-Derived Reactive Oxygen Species Play an Important Role in Doxorubicin-Induced Platelet Apoptosis	Chemotherapy Agent (Doxorubicin); Oxidative Stress	Article
Wang *et al.* [22]	Abnormal Mitochondrial Function and Impaired Granulosa Cell Differentiation in Androgen Receptor Knockout Mice	Androgen Receptor; Reproduction	Article
